# EHMTI-0370. The performance of the ID-Migraine questionnaire in a Hungarian sample

**DOI:** 10.1186/1129-2377-15-S1-J3

**Published:** 2014-09-18

**Authors:** E Csepany, M Toth, D Janoska, I Kellermann, B Hajnal, T Gyure, G Bozsik, C Ertsey

**Affiliations:** 1 János Szentágothai Neurosciences PhD School, Semmelweis University, Budapest, Hungary; 2 Headache Service, Vaszary Kolos Hospital, Esztergom, Hungary; 3 Faculty of Medicine, Semmelweis University, Budapest, Hungary; 4 Department of Neurology, Semmelweis University, Budapest, Hungary

## Background

Migraine causes significant disability and severely affects patients' quality of life. A significant proportion of patients is not diagnosed, and therefore not treated adequately. A short and reliable migraine screening tool could improve disease identification. ID-Migraine is a simple and reliable screening tool, originally developed in the United States, but also used in other countries and languages.

## Objective

To assess the performance of the ID-Migraine questionnaire on a Hungarian sample.

## Methods

Outpatients at the Headache Service, Department of Neurology, Semmelweis University completed the Hungarian version of ID-Migraine. The gold standard was the clinical diagnosis made by a headache specialist according to the ICHD criteria. We calculated the sensitivity, specificity, positive and negative predictive value and the misclassification rate.

## Results

305 patients (241 females, mean age 39,2±13,3 years) were enrolled. The diagnosis was migraine in 244, tension type headache in 37, cluster headache in 16 and other headaches in 8. The questionnaire's sensitivity was 0.95 and specificity was 0.56. The positive predictive value was 0.89 and the negative predictive value was 0.78. The misclassification rate was 0.13.

## Discussion

The Hungarian version of ID-Migraine was easy to use and well accepted by patients. Its specificity was somewhat lower than that of the original questionnaire; its other measures of performance were similar to those of the original version and its existing Italian, Turkish and Portuguese translations. ID-Migraine seems to be a promising screening tool for migraine in the Hungarian population.

No conflict of interest.

